# Retinoic Acid Signalling and the Control of Meiotic Entry in the Human Fetal Gonad

**DOI:** 10.1371/journal.pone.0020249

**Published:** 2011-06-03

**Authors:** Andrew J. Childs, Gillian Cowan, Hazel L. Kinnell, Richard A. Anderson, Philippa T. K. Saunders

**Affiliations:** 1 Medical Research Council Human Reproductive Sciences Unit, The Queen's Medical Research Institute, University of Edinburgh, Edinburgh, United Kingdom; 2 Division of Reproductive and Developmental Sciences, Centre for Reproductive Biology, The Queen's Medical Research Institute, University of Edinburgh, Edinburgh, United Kingdom; McGill University, Canada

## Abstract

The development of mammalian fetal germ cells along oogenic or spermatogenic fate trajectories is dictated by signals from the surrounding gonadal environment. Germ cells in the fetal testis enter mitotic arrest, whilst those in the fetal ovary undergo sex-specific entry into meiosis, the initiation of which is thought to be mediated by selective exposure of fetal ovarian germ cells to mesonephros-derived retinoic acid (RA). Aspects of this model are hard to reconcile with the spatiotemporal pattern of germ cell differentiation in the human fetal ovary, however. We have therefore examined the expression of components of the RA synthesis, metabolism and signalling pathways, and their downstream effectors and inhibitors in germ cells around the time of the initiation of meiosis in the human fetal gonad. Expression of the three RA-synthesising enzymes, *ALDH1A1*, *2* and *3* in the fetal ovary and testis was equal to or greater than that in the mesonephros at 8–9 weeks gestation, indicating an intrinsic capacity within the gonad to synthesise RA. Using immunohistochemistry to detect RA receptors RARα, β and RXRα, we find germ cells to be the predominant target of RA signalling in the fetal human ovary, but also reveal widespread receptor nuclear localization indicative of signalling in the testis, suggesting that human fetal testicular germ cells are not efficiently shielded from RA by the action of the RA-metabolising enzyme CYP26B1. Consistent with this, expression of *CYP26B1* was greater in the human fetal ovary than testis, although the sexually-dimorphic expression patterns of the germ cell-intrinsic regulators of meiotic initiation, *STRA8* and *NANOS2*, appear conserved. Finally, we demonstrate that RA induces a two-fold increase in *STRA8* expression in cultures of human fetal testis, but is not sufficient to cause widespread meiosis-associated gene expression. Together, these data indicate that while local production of RA within the fetal ovary may be important in regulating the onset of meiosis in the human fetal ovary, mechanisms other than CYP26B1-mediated metabolism of RA may exist to inhibit the entry of germ cells into meiosis in the human fetal testis.

## Introduction

Primordial germ cells (PGCs) are the embryonic precursors of sperm and egg in the adult organism. Although initially bipotential, with the capacity to adopt spermatogenic or oogeneic fates, the developmental trajectory of PGCs is dictated by the somatic sex of the gonad in which they find themselves following migration [1]. PGCs which find themselves in a female (ovarian) environment enter meiosis from embryonic day (e)13.5 in the mouse and 11 weeks gestation in the human, whilst germ cells in the developing testis progressively enter a state of cell cycle arrest, resuming proliferation and differentiation only after birth [2].

The mechanism(s) by which this dimorphism in meiotic entry is established has long been a matter of debate. Recent data have suggested that meiosis is initiated in the fetal mouse ovary by retinoic acid (RA) signalling, which acts on germ cells to promote the expression of Stimulated by Retinoic Acid (*Stra8*) [3], [4], a protein required for pre-meiotic DNA replication [5]. Germ cells in the fetal mouse testes are shielded from the meiosis-inducing action of RA, first by somatic cell expression of the RA-metabolising enzyme Cyp26b1 [3], [4], then subsequently by the action of the germ cell-expressed RNA-binding protein Nanos2, which acts to ‘lock in’ the male germ cell differentiation program and repress *Stra8* expression in testicular germ cells following the downregulation of Cyp26b1 [6].

RA is a potent morphogen that exerts diverse effects during development and differentiation [7], [8], [9]. It is tightly regulated by a group of RA synthesising- and metabolising-enzymes. The retinaldehyde dehydrogenase enzymes (Aldh1a 1,2 and 3) are responsible for the oxidation of RA precursors to produce RA [10], [11], while RA signalling is negatively regulated by three RA degrading enzymes, Cyp26A1, Cyp26B1 and Cyp26C1, which metabolize RA into hydroxylated polar derivatives [12]. Although undetectable in the fetal gonad itself, expression of the RA synthesis enzymes *Aldh1a2* and *Aldh1a3* has been demonstrated in mesonephroi of fetal mice between e11.5 and 13.5, and the mesonephros has been shown to synthesis high levels of RA at this stage [3]. A source-sink model of meiotic entry in the fetal mouse gonad has therefore been proposed [3], [4], in which the delivery of mesonephros-derived RA into the anterior end of the gonad, and its subsequent diffusion along the gonadal axis, results in the entry of germ cells in the fetal ovary into meiosis in a rostro-caudal (anterior-posterior) wave, with expression of PGC/pluripotency-associated markers such as *Oct4* downregulated [13], [14], [15], and markers of meiosis such as *Stra8*, Synaptonemal Complex Protein 3 (*Sycp3*) and the Dosage suppressor of *mck1* homologue 1 (*Dmc1*) [14], [15] upregulated.

In contrast to the rodent however, germ cell differentiation in the human fetal ovary does not occur in a synchronized rostro-caudal wave. Germ cells at different developmental stages are instead arranged radially, with undifferentiated PGC-like OCT4-positive/VASA-negative germ cells present at the periphery of the organ and more differentiated OCT4-negative/VASA-postitive germ cells deeper within the medulla [16], [17]. Significantly, these subpopulations exist in parallel, overlapping in space and time such that undifferentiated OCT4-positive cells are still detectable at the periphery of the ovary several weeks after the first germ cells enter meiosis, and even beyond the onset of follicle formation [16]. This suggests that local control of germ cell differentiation may play a greater role in the human fetal ovary than occurs in the rodent at the equivalent developmental stage, a hypothesis supported by the recent demonstration of intrinsic RA synthesis and metabolism by the human fetal ovary [18].

The aim of this study was to determine whether similar mechanisms to those reported to control the initiation of meiosis in mouse fetal germ cells also operate to control this process in the human fetal gonad, by examining the expression and localization of key components of the retinoid synthesis, signalling and effector machinery in the human fetal testis and ovary across the period of meiotic initiation. We report differences in the expression and localization of RA metabolising enzymes in the developing human gonad to those reported in mice at similar developmental stages, notably greater expression of *CYP26B1* in the ovary compared to the testis. We find germ cells to be the primary target of retinoid signalling in the human fetal ovary, but reveal RA receptor expression – and activation – to be widespread in the human fetal testis, indicating that RA metabolism does not fully shield human fetal testicular cells from RA signalling activity. Finally, we demonstrate that RA has the capacity to induce *STRA8* expression in the human fetal testis, but does not increase expression of other genes associated with the initiation of meiosis. Together these data suggest that the control of meiotic initiation in the human fetal ovary may not be controlled exclusively by retinoid signalling and metabolism, and that there may be greater emphasis on the regulation of meiosis at a local, rather than whole-organ level in the human fetal ovary, than occurs in mouse.

## Results

### Expression of the genes encoding retinaldehyde dehydrogenase enzymes during human fetal gonadal development

The mesonephros of the developing mouse embryo is thought to be the site of synthesis of meiosis-inducing RA, and mesonephric (but not gonadal) expression of the genes encoding RA synthesising enzyme (Aldh1a2 and Aldh1a3) has been reported [3]. We examined the expression of RA synthesis enzymes *ALDH1A1*, *ALDH1A2* and *ALDH1A3* at three gestational stages (namely 8–9, 14–16 and 17–20 weeks gestation) which broadly reflect the timing of key events in the development of the human fetal gonad. At 8–9 weeks gestation, sex determination has occurred, yet male and female gonads contain only undifferentiated proliferating primordial germ cells. By 14–16 weeks, meiosis has initiated and syncitial germ cell nests are forming alongside ongoing germ cell proliferation in the fetal ovary. Finally, at 17–20 weeks, germ cell nests in the fetal ovary start to break down, primordial follicle formation commences and germ cells become arrested at the diplotene stage of meiotic prophase I. A progressive process of germ cell maturation also occurs in the fetal testis during this period, with a progressive loss of primordial germ cell-associated marker expression [16], [19].

**Figure 1:**
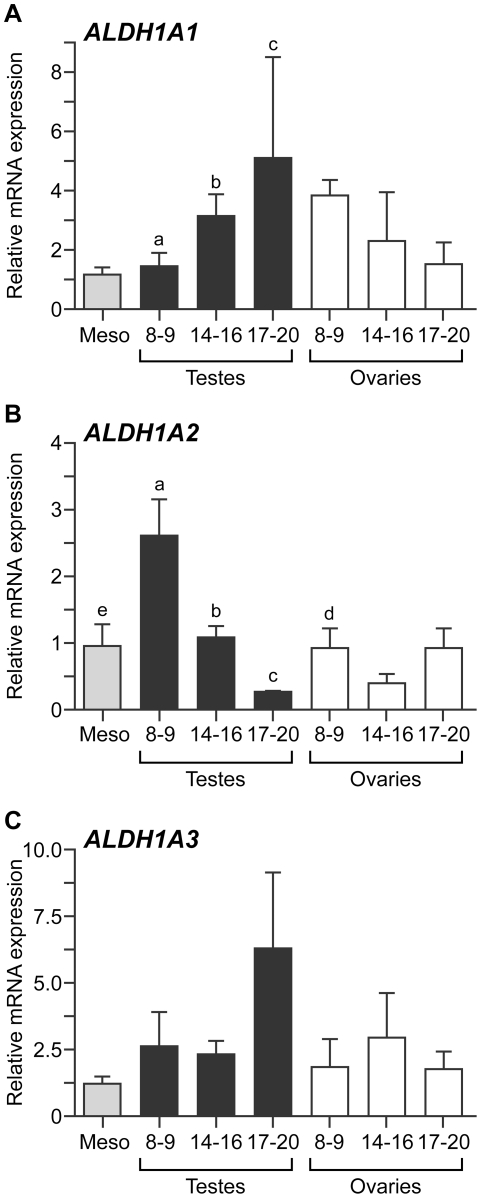
Expression of genes encoding retinaldehyde dehydrogenase enzymes in the human fetal gonad. qRT-PCR analysis reveals developmentally regulated expression of *ALDH1A1* (A) in the human fetal testis, with transcript levels increasing significantly between 8–9 weeks gestation and 14–16/17–20 weeks gestation (ANOVA; a,b,c; p<0.05, n = 5–6 per group). Expression was not significantly different between gonads of different sexes at the same developmental stage, not between ovaries at different gestational ages. *ALDH1A2* expression (B) is also developmentally-regulated in the human fetal testis, with transcript levels in the first trimester (8–9 weeks gestation) testis significantly higher than those in the early second trimester (14–16 weeks) testis (a vs b, p<0.05) and the late second trimester (17–20 weeks) testis (a vs c, p<0.01). Expression in the testis at 8–9 weeks gestation was also significantly higher than that in the fetal ovary at the same developmental stage (a vs d, p<0.05). *ALDH1A2* transcript levels were also higher in the 8–9 week human fetal testis than in mesonephroi from age-matched fetuses (a vs e, p<0.01), which contrasts with the mesonephric-specific expression of *Aldh1a2* in the mouse at a comparable developmental stage. No differences in the expression of *ALDH1A3* (C) were detected between samples of different gestational ages of the same sex, or between the gonads of different sexes at the same developmental stage. 8–9, 14–16 and 17–20 denote the gestational age (in weeks) of specimens, meso: 8–9 week mesonephroi (pooled male and female). Values denote mean ± s.e.m..

In the human fetal testis, *ALDH1A1* expression increased significantly across the gestational range examined (p<0.05; Figure 1A). In contrast, there was a trend towards decreasing expression of *ALDH1A1*in the fetal ovary over the same period (Figure 1A) although this did not reach significance. We found no significant differences in expression of *ALDH1A1* between gonads of different sexes of the same gestational age, or between fetal gonads and mesonephroi from 8–9 week fetuses. *ALDH1A2* expression in the fetal testis was significantly higher at 8–9 weeks gestation than at 14–16 weeks (p<0.05) or 17–20 weeks gestation (p<0.01. Figure 1B). Furthermore, at 8–9 weeks gestation we detected a sexual dimorphism in *ALDH1A2* expression, with transcript levels significantly higher in the testis than the ovary at this developmental stage (p<0.05). Notably, *ALDH1A2* transcript levels were also higher in the 8–9 week human fetal testis than in mesonephroi from the same fetuses (p<0.01), indicating that in humans the gonad, rather than the mesonephros, may be the predominant site of RA synthesis. No significant differences in the expression of *ALDH1A3* were detected either within or between sexes at any developmental stage (Figure 1C) although, as with *ALDH1A1* and *ALDH1A2*, expression in both ovary and testis was not lower than in mesonephros. Together, these data support the hypothesis [18] that the human fetal gonad has an intrinsic capacity to produce retinoic acid.

### Expression and localisation of retinoid receptors in the human fetal gonads

Retinoid signals are transduced by two families of receptors, RAR and RXR receptors, which can hetero- and homodimerise to regulate gene expression. Inhibition of RA receptor action has been shown to inhibit meiosis in RA-treated mouse fetal testes [3], [4] and in the human fetal ovary [18]. To identify the possible receptors involved in retinoid signalling in the human fetal gonad, we examined the expression of the RA receptors (RARα, β and γ) and the retinoid receptors (RXRα, β and γ) at the transcript and protein level in the developing human fetal ovary and testis.

**Figure 2:**
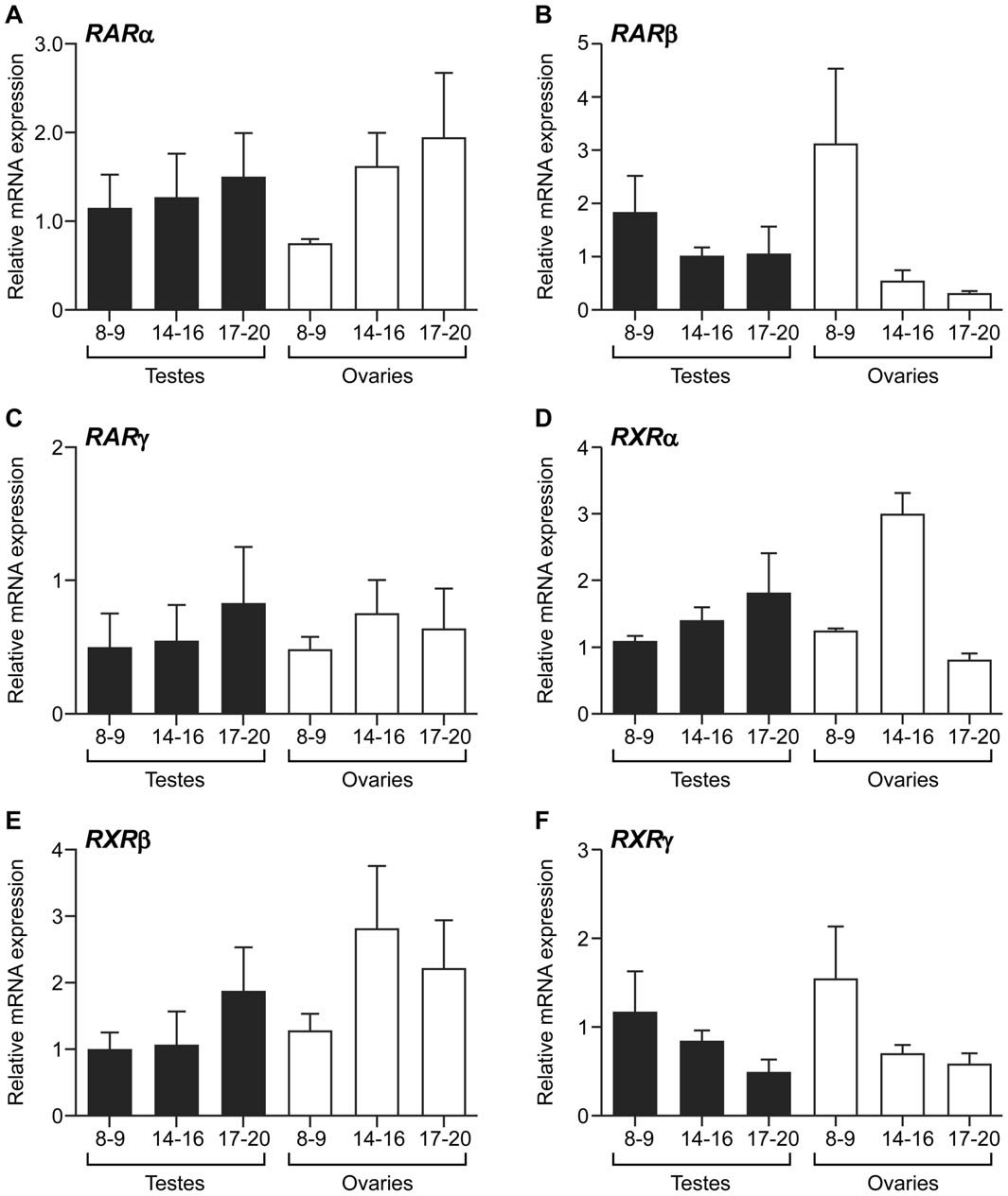
Expression of genes encoding retinoic acid and retinoid receptors in the human fetal gonad. qRT-PCR analysis of expression of the genes encoding the retinoic acid (RARα (A), RARβ (B) and RARγ (C) and retinoid (RXRα (D), RXRβ (E) and RXRγ (F)) in the human fetal testis and ovary. No significant differences in levels encoding any of the receptor isoforms were detected between samples of different gestational ages within the same sex, or in gonads of different sexes at the same developmental age, indicating that RAR/RXR receptor expression is not developmentally-regulated in the human fetal gonad. Values denote mean ± s.e.m; 8–9, 14–16 and 17–20 denotes gestational age (in weeks) of specimens.

We detected transcripts encoding all three RAR (Figure 2A–C) and RXR (Figure 2D–F) receptors in human fetal testes and ovaries. Interestingly however, we did not detect any significant changes in expression of any of the receptor isoforms either between gonads obtained from fetuses of the same sex at different gestational ages, or between those of different sexes at the same developmental stage (Figure 2A–F, n = 4–6 per group). Expression of the retinoid receptor machinery therefore appears not to be developmentally-regulated at the transcript level in human fetal gonads around the time of meiosis.

**Figure 3:**
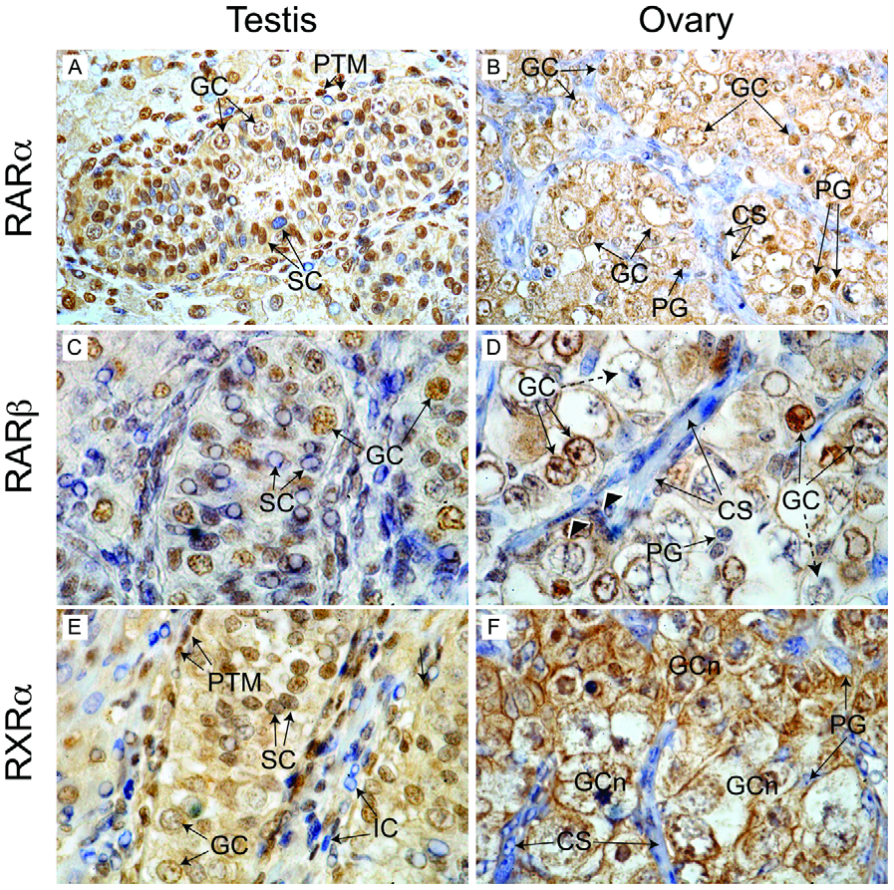
Immunohistochemical localisation of retinoid receptor expression in the human fetal gonad. In the second trimester human fetal testis (A) RARα staining was detected in germ cell (GC) and peritubular myoid (PTM) nuclei. Two populations of Sertoli cells (SC; immunopositive and immunonegative) could also be detected. In the fetal ovary at the same developmental stage (B), RARα expression was detected in the nuclei and cytoplasm of germ cells in nests, and in the nuclei of pregranulosa cells (PG) interspersed between germ cells. Mesenchymal somatic cells in streams (CS) displayed variable staining. RARβ expression was widespread in the fetal testis (C) with all major cell populations displaying intense nuclear staining. In contrast, variable RARβ expression was detected in the germ cells of the fetal ovary (D); with some displaying intensely stained nuclei or both nuclear and cytoplasmic staining (solid arrows) and others showing little or no staining (dashed arrows). Pregranulosa cells were immunonegative, as were somatic cells in streams, although some displayed nuclear staining for RARβ (arrowheads). Peritubular myoid and Sertoli cell nuclei in the testes displayed intense staining for RARβ (E), with weaker expression in detected in germ cells. A population of immunonegative IC was also detected. The distribution of immunostaining for RXRα in the fetal ovary (F) was comparable to that of RARβ, with expression restricted to germ cells in nests (GCn) and absent in somatic cell streams and pregranulosa cells. The widespread nuclear localization of RA receptors in testis suggests cells of all types (including germ cells) are exposed to RA signals. Magnification: 400× (A, B), 1000× (C–F).

To establish the cellular targets of retinoid signalling in the human fetal testis and ovary, we performed immunohistochemistry using specific antibodies raised against RARα, RARβ and RXRα on sections of second trimester human fetal ovaries and testes (Figure 3). RARα expression was widely distributed in the second trimester human fetal testis (Figure 3A). Expression was detected in germ cells, which displayed either nuclear or both nuclear and cytoplasmic staining. Sertoli cells were predominantly immunopositive, and displayed strong nuclear staining, although a sub-population of these cells could be identified which did not express RARα. Peritubular myoid (PTM) cells were also mostly immunopositive, with the nucleus the predominant site of receptor localisation in this cell type. Interstitial cells were mostly immunopositive, although a subpopulation which showed no staining was also detectable. RARα expression in the human fetal ovary at a comparable developmental stage was present in germ cells in syncitial clusters (also known as germ cell nests; Figure 3B), and localised to both the cytoplasm and nuclei of these cells. Pregranulosa cells interspersed between germ cells also displayed strong nuclear staining for RARα. Expression of RARα in streams of mesenchymal cells between germ cell nests was variable, with some cells displaying nuclear staining and some being immunonegative.

Like RARα, the expression of RARβ in the second trimester testis was broadly distributed, with germ, Sertoli, PTM and interstitial cells all immunopositive (Figure 3C). In contrast to RARα however, the subcellular localisation of RARβ appeared to be predominantly nuclear in all cell types examined. In the fetal ovary, germ cells in syncitial clusters were again the predominant site of RARβ expression, with a mixture of large weakly-staining germ cells in which staining was cytoplasmic, and smaller germ cells with intensely immunopositive-nuclei (Figure 3D). Pregranulosa cells displayed no staining, whilst RARβ staining was either weak or absent in the nuclei of mesenchymal somatic cells.

In the fetal testis, Sertoli and PTM cells displayed intense nuclear staining for RXRα (Figure 3E). Germ cells also expressed this receptor, but displayed weaker staining in both the cytoplasm and nucleus. A subpopulation of interstitial cells with immunonegative nuclei could also be discerned. In the fetal ovary, RXRα expression was similar to that of RARβ, with germ cells in nests displaying both nuclear and cytoplasmic staining (Figure 3F), whilst mesenchymal somatic cells and pregranulosa cells were immunonegative.

These data suggest that RA and its derivatives likely target a diverse range of cell types in the second trimester human fetal testis, whilst germ cells are the predominant target of retinoid action in the fetal ovary at a comparable developmental stage.

### Expression of genes encoding *STRA8*, *CYP26B1* and *NANOS1-3* in the developing human fetal gonad

In the fetal mouse ovary, RA induces the expression of Stra8, which is required for pre-meiotic DNA replication and progression through meiosis [5]. Germ cells in the fetal testis are shielded from the meiosis-inducing action of RA by Sertoli cell-expression of the RA metabolising enzyme Cyp26b1, and later by the expression of Nanos2 in germ cells which acts to ‘lock in’ the male fate. As conservation of the expression patterns of these genes in the human fetal gonad is likely to reflect functional conservation with their roles in mice, we compared the expression of *STRA8*, *CYP26B1* and *NANOS2* (and its paralogues *NANOS1 and NANOS3*) in the male and female human fetal gonad across the developmental window encompassing meiotic entry outlined above.

**Figure 4:**
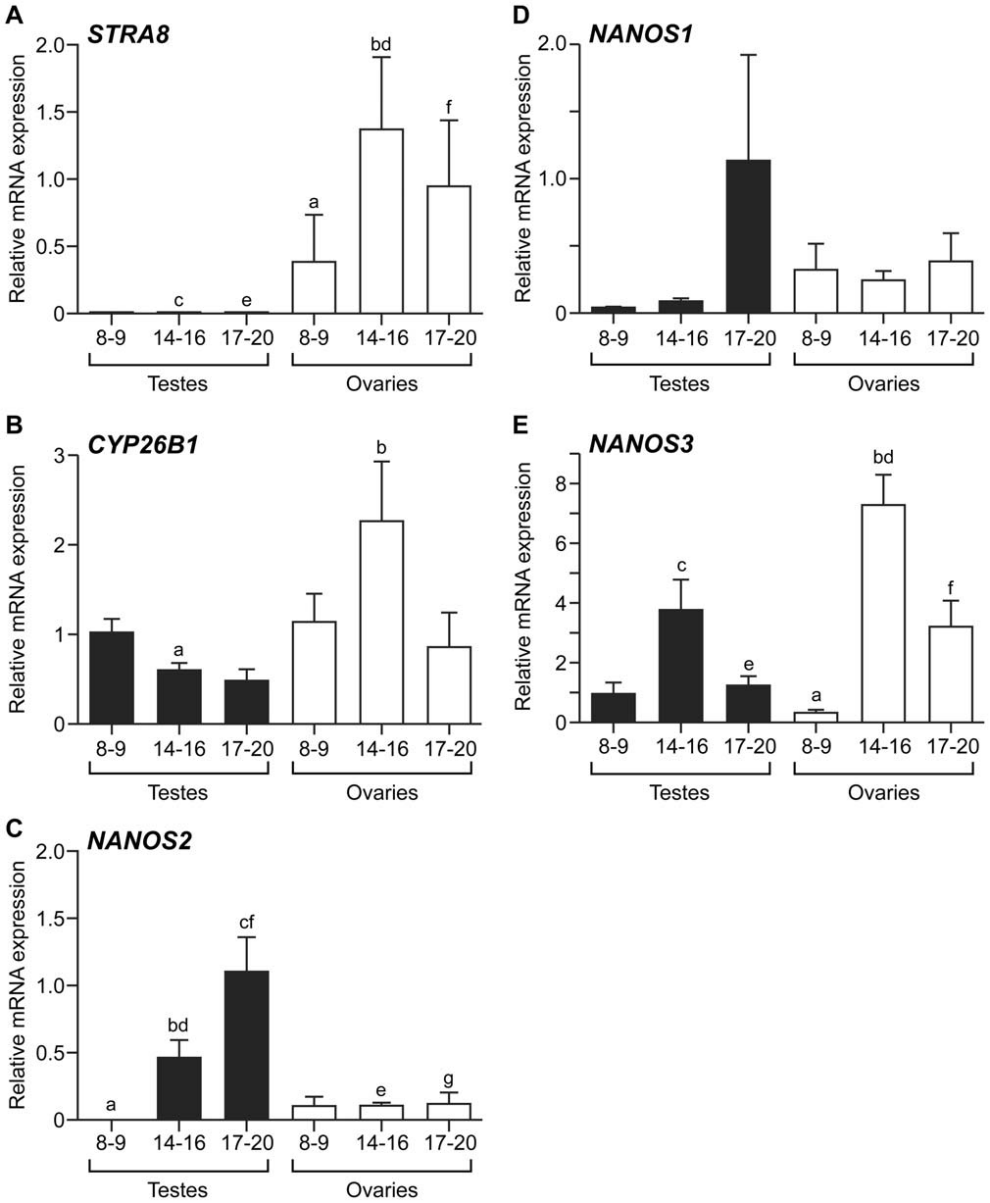
Conserved and divergent patterns of expression of *STRA8*, *CYP26B1* and *NANOS1-3* in the human fetal gonad. qRT-PCR analysis of human fetal gonads reveals female-biased and developmentally-regulated expression of *STRA8* (A). *STRA8* expression increased significantly between 8–9 and 14–16 weeks in the human fetal ovary (a vs b, p<0.05) consistent with the initiation of meiosis in the fetal ovary around 11 weeks gestation. Levels of transcripts encoding *STRA8* were low and not significantly different between human fetal testes and ovaries at 8–9 weeks gestation, but were significantly higher in fetal ovaries than fetal testes at 14–16 weeks (c vs d, p<0.0001) and 17–20 weeks (e vs f, p = 0.008). *CYP26B1* (B) expression was not significantly different between samples of the same sex at different gestational ages, but was significantly higher at in the fetal ovary than the fetal testis at 14–16 weeks (a vs b, p = 0.02); suggesting the male-specific expression of *CYP26B1* reported in mice at a comparable developmental stage is not conserved to humans. *NANOS2* (C) expression was predominantly male-specific and developmentally-regulated, with expression increasing in the human fetal testis with increasing gestational age (a,b,c, p<0.001). *NANOS2* expression was also significantly higher in fetal testes than ovaries at 14–16 weeks (d vs e, p = 0.01) and 17–20 weeks (f vs g, p<0.01), a result consistent with a role for this protein in repressing meiosis in the fetal male germline. 8–9, 14–16 and 17–20 denote gestation age (in weeks) of specimens. No differences were detected in the expression of *NANOS1* (D) between testis and ovaries at any gestational age, nor between gonads of the same sex at any developmental stage. *NANOS3* expression (E) was significantly higher in the human fetal ovary at 14–16 weeks gestation than at 8–9 weeks gestation (a vs b, p<0.05), in contrast to the downregulation of the homologous gene in the fetal mouse ovary at the comparable developmental stage. Expression of *NANOS3* was also greater in the fetal ovary than in the fetal testis at 14–16 weeks (c vs d, p<0.05) and 17–20 weeks (e vs f, p<0.02). 8–9, 14–16 and 17–20 denote the gestational age (in weeks) of specimens, values denote mean ± s.e.m, 8–9, 14–16 and 17–20 denote the gestational age (in weeks) of specimens.

Expression of *STRA8* was low/absent in the gonads of both sexes at 8–9 weeks gestation (Figure 4A), consistent with these tissues containing only pre-meiotic proliferating germ cells at this developmental stage. *STRA8* expression increased significantly between 8–9 and 14–16 weeks in the human fetal ovary however (p<0.05), concomitant with the initiation of meiosis in this tissue. STRA8 expression remained very low in the testis at all gestations examined, and was significantly higher in the fetal ovary than testis at 14–16 weeks (p<0.0001) and 17–20 weeks (p<0.008) gestation. The developmental and sex-specific pattern of *Stra8* expression therefore appears to be conserved between the fetal gonads of humans and mice at comparable developmental stages, as reported previously [18], [20].

In contrast to *STRA8* however, the expression of *CYP26B1* in the human fetal gonad diverges significantly from that previously reported in mouse. *CYP26B1* expression was not significantly different between ovaries and testes at 8–9 weeks gestation, but was unexpectedly higher in 14–16 week ovaries than testis (p = 0.02, Figure 4B). To determine whether another member of the CYP26 family may be responsible for retinoid metabolism in the human fetal testis, we also examined the expression of *CYP26A1* and *CYP26C1*, but were unable to detect transcripts for either gene at any gestational age examined in both fetal testis and ovary (data not shown). These data suggest that the ovary may have a greater capacity for RA metabolism than the fetal testis at this stage.

*NANOS2* displayed both developmentally-regulated and sexually-dimorphic expression in the human fetal gonad (Figure 4C). *NANOS2* expression was significantly higher in the fetal testis at 17–20 weeks gestation than at 8–9 weeks, consistent with the progressive commitment of germ cells to the male fate between these developmental stages. Whilst no difference in *NANOS2* expression was detected between testes and ovaries at 8–9 weeks gestation, *NANOS2* transcript levels were significantly higher in the fetal testis than ovary at 14–16 weeks (p = 0.01) and 17–20 weeks (p<0.01). The expression of *NANOS2* in the human fetal testis therefore appears to mirror that reported for the homologous gene in mouse, indicating possible conservation of its functional role in reinforcing the male fate in testicular germ cells between these two species.

We also examined the expression of the related genes *NANOS1* and *NANOS3* in the developing fetal gonad. We found no differences in the expression of *NANOS1* between testes and ovaries at any gestational age, nor between gonads of the same sex at different developmental stages (Figure 4D). Unexpectedly however, *NANOS3* transcript levels were found to be significantly higher in ovaries than testes at 14–16 weeks (p<0.05) and at 17–20 weeks gestation (p = 0.02; Figure 4E), revealing a sexual dimorphism in expression levels in this gene that has not previously been reported in mice. Strikingly – and in stark contrast to the expression of *Nanos3* in the fetal mouse ovary, which is downregulated shortly after the entry into meiosis from e14.5 onwards - the expression of *NANOS3* increased significantly between 8–9 and 14–16 weeks gestation in the human fetal ovary (p<0.05), suggesting a possible role in meiosis for this protein.

### Retinoic acid induces *STRA8* expression in the second trimester human fetal testis, but does not affect the expression of other meiosis-associated genes

**Figure 5:**
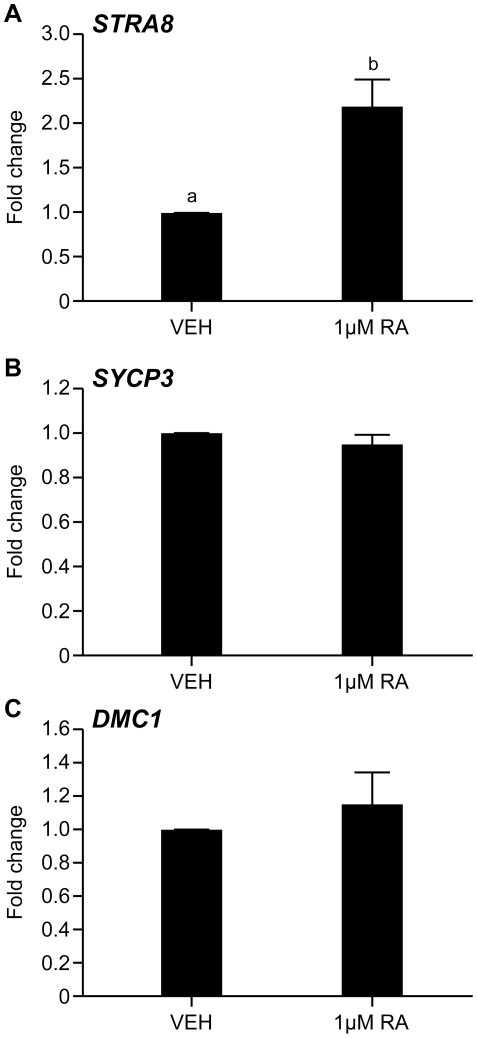
Retinoic acid promotes the expression of *STRA8* but not other meiosis-associated genes in the second trimester human fetal testis. Human fetal testis (14–15 weeks gestation) were disaggregated to a single cell suspension and cultured in serum free medium in the presence of vehicle (DMSO) or 1 µM all *trans* retinoic acid (1 µM RA in DMSO) for 24 hours. RA treatment induced a 2.2±0.3 fold increase in the expression of *STRA8* (A) relative to vehicle treated controls (a vs b, p<0.05, n = 5), indicating that RA-regulated expression of this gene is conserved in human fetal gonads. No significant differences in the expression of meiosis markers *SYCP3* (B, n.s. n = 6) or *DMC1* (C, n = 6, n.s.) were detected between RA- and vehicle-treated cultures, indicating that whilst RA can stimulate *STRA8* expression, it is not sufficient to cause widespread activation of the meiosis-associated gene expression programme. Values denote mean ± s.e.m..

To determine whether the induction of *STRA8* expression by RA is conserved between mouse and human, we investigated the effects of RA treatment on cells from the second trimester human fetal testis. To ensure germ cell exposure to RA, fetal testes (14–15 weeks gestational age, n = 6) were disaggregated to a single cell suspension (thus uncoupling germ cells from their associated somatic cells which are in the mouse thought to be the site of CYP26B1 expression) and cultured in serum free medium in the presence of either vehicle (DMSO) or 1 µM all-trans RA for 24 hours. Expression of *STRA8*, and of the meiosis markers *SYCP3* and *DMC1* was then assessed by qRT-PCR in control and RA-treated cultures. Treatment with RA for 24 hours resulted in a 2.2±0.3 fold increase in *STRA8* expression relative to vehicle-treated controls (p<0.05, n = 5; Figure 5A); revealing conserved induction of *STRA8* expression by RA in the human fetal testis. No significant differences were found between RA- and vehicle treated cultures in the expression of *SYCP3* (94.4±5.7% of controls, n.s; Figure 5B) or *DMC1* (115.1±19.6% of controls, n.s.; Figure 5C) however, suggesting that whilst RA can selectively induce the expression of *STRA8* in the human fetal testis, it may not be sufficient to induce additional meiosis-associated gene expression at this developmental stage, or in this experimental system.

## Discussion

In the fetal mouse gonad, germ cell differentiation proceeds in a rostro-caudal wave, possibly reflecting the diffusion of mesonephros-derived retinoic acid along the long axis of the gonad [3], [4], [14], [15]. Whilst meiosis is also thought to initiate at the cranial end of the fetal ovary in the human [21], within weeks a radial distribution of germ cells is detectable, with undifferentiated pre-meiotic PGC-like cells found at the periphery of the ovary and progressively more differentiated germ cells found towards the central cortex [16], [17]. The existence of multiple subpopulations of germ cells at different developmental stages within the human fetal ovary indicates that differentiation is far less synchronous than in the mouse, and raises the question as to how this asynchrony is maintained. In this report we have examined the expression of key components of the retinoid synthesis and signalling apparatus in the human fetal gonad, and of key downstream effectors (*STRA8*) and antagonists (*CYP26B1*, *NANOS2*) of the meiosis-inducing action of RA. Whilst identifying conservation of some aspects of the regulation of meiotic entry between mice and humans, we have also identified significant species-specific differences in the expression of genes associated with the entry or inhibition of meiosis, which may contribute to or help explain the differing spatiotemportal organization of germ cell differentiation in the fetal ovary. Our findings also support the recent hypothesis that intrinsic RA synthesis within the ovary, rather than RA originating from the mesonephros, may be the primary driver of meiotic initiation in the human fetal ovary [18].

The genes encoding the retinoid synthesis enzymes Aldh1a2 and Aldh1a3 are expressed in the mesonephroi of the fetal mouse around the time of the initiation of meiosis, but their expression is undetectable within the gonad itself [3]. In this report however, we demonstrate the expression levels of all three *ALDH1A* genes in the gonads of both sexes to be at least equal to those detected in mesonephroi from fetuses at 8–9 weeks gestation, suggesting that unlike that of the mouse, the human fetal gonad has an intrinsic capacity to synthesis RA. We found the expression of *ALDH1A2* to be sexually-dimorphic, being significantly greater in the fetal testis at 8–9 weeks gestation than in ovaries from age matched samples or compared to testis from fetuses at later gestational ages. We found no significant differences in the level of expression of *ALDH1A1* between the ovary and testis however, in contrast to the male-specific expression of *Aldh1a1* reported in the fetal mouse and chicken at a comparable developmental stage [22]. The male-biased expression of *ALDH1A2* in the human fetal testis may replace the function of *Aldh1a1* in these species. Our data also both compliment and contrast with that reported recently by Le Bouffant *et al.*, who found the expression of *ALDH1A1* to increase sharply in the ovary around 11 weeks post-fertilization (wpf), coincident with the onset of meiosis (yet was lower in all ovarian samples examined than the 9 wpf testes), whilst *ALDH1A2* expression was relatively stable across the developmental range examined [18]. A direct comparison of our data with that of Le Bouffant *et al.* [18] is not straightforward, given the different systems of aging fetal specimens, the range of gestational ages examined and the number of independent biological replicates (fetal specimens) used for each time point examined. Despite these specific differences however, the data reported here and by Le Bouffant *et al.* [18] support the same conclusion; that unlike the mouse, the fetal human gonad has an intrinsic capacity to produce RA, and thus gonadal, rather than mesonephric RA synthesis may drive the initiation of meiosis in germ cells in the human fetal ovary. Furthermore, local production rather than diffusion of RA from the mesonephros may help explain the asynchronous entry of germ cells into meiosis in the human fetal gonad.

We have examined for the first time the expression of all of the genes encoding the RAR and RXR retinoid receptor proteins during human fetal gonadal development, and demonstrated protein expression of members of both receptor families (RARα, RARβ and RXRγ) in the germ cells of the fetal human ovary, and in a wide range of cell types in the human fetal testis. Our finding that germ cells in the human fetal ovary are transducing RA signals (as indicated by nuclear localization of the receptors) contrasts with that reported by Morita and Tilly in the fetal mouse ovary at a comparable developmental stage (e13.5), who (using pan-RAR and pan-RXR antibodies) reported only weak cytoplasmic expression of RAR proteins, and – in contrast to the data presented here - no expression of the RXR proteins [23]. In the same study, treatment of e13.5 fetal mouse ovaries with a relatively low concentration of RA (0.01 µM) resulted in the relocalisation of RAR proteins to germ cell nuclei, leading the authors to conclude that RA signalling must either be extremely low or absent in the fetal ovary *in vivo* at this time [23]. The absence of nuclear-localised RA receptors in the fetal mouse ovary around the time of the initiation of meiosis seemingly contradicts the model in which RA signalling in ovarian germ cells around e13.5 stimulates *Stra8* transcription and subsequent meiotic entry, but suggests perhaps that only very low levels of RA (i.e insufficient to cause widespread receptor nuclear localization) are required to initiate meiosis in the fetal mouse ovary. The predominantly nuclear localization of RARs in human ovarian fetal germ cells may indicate the existence of higher local RA concentrations than are present in the mouse fetal ovary, perhaps arising from intrinsic ovarian, rather than mesonephric RA synthesis. Alternatively, it may reflect a broader role for RA signalling in germ cell development in the human beyond the regulation of meiotic entry, such as in the regulation of germ cell survival or proliferation [18].

The most significant aspect of the immunohistochemical analyses reported here however, is the identification of germ cells displaying nuclear staining for RARα, RARβ and RXRα in the human fetal testes. This strongly suggesting that they are both receiving and transducing retinoid signals and are therefore not effectively shielded by the action of the somatic cell-expressed metabolizing enzyme CYP26B1 as is believed to be the case in the fetal mouse testis; [3], [4]. The widespread localization of the RA receptors in the human fetal testis also contrasts with reports that RARα and RARβ are undetectable in the developing rodent testis prior to e16 [24], providing further evidence of extensive divergence in testicular RA signalling between these species.

These data, coupled with greater expression of *CYP26B1* expression in the human fetal ovary than testis (this paper and [18]; again in contrast to the male-specific expression of *Cyp26b1* in the fetal mouse testes), suggests a less important role for CYP26B1 in the regulation of meiosis in the developing gonad in the human than in the mouse. Although the inappropriate entry of testicular germ cells into meiosis in mice homozygous for targeted disruptions of *Cyp26b1* [25], [26], and in *in vitro* cultures of fetal testes in which Cyp26b1 is inhibited with ketoconazole [3], [4] provide compelling evidence of a role for Cyp26b1 in inhibiting the initiation of meiosis in testicular germ cells, aspects of this model have recently come under increasing scrutiny. The ketoconazole culture experiments have not been replicated by other groups [27], and ketoconazole is unable to ameliorate the inhibitory effects on ovarian germ cell meiotic entry of a testis co-cultured with an ovary [28], as would be expected if Cyp26b1 metabolism of RA was the key inhibitor of meiotic initiation. Together, these data argue that other mechanisms that inhibit the entry of germ cells into meiosis in the fetal testis are likely to exist. The recent identification of FGF9 as an inhibitor of RA-induced meiosis in the fetal mouse testis [29], and the demonstration that secretory pathways and their cargoes play a key role in determining germ cell sex determination [27], [28] provide further evidence for the existence of additional mechanisms that inhibit meiotic entry in the fetal testis, or conversely promote it in the ovary.

Some aspects of meiosis initiation and germ cell sex determination do appear to be conserved between mouse and human, however. We find *STRA8* expression to increase between 8–9 and 14–16 weeks gestation, consistent with previous reports of the expression of this gene increasing around the onset of meiosis in the human fetal ovary [18], [20] and reflecting a comparable increase in *Stra8* expression in the fetal mouse ovary from e13.5 onwards. We have also conducted the first comparative analysis of gene expression of the *NANOS* family in the developing human fetal ovary and testis. We find the expression of *NANOS2* to be restricted to the human fetal testis, consistent with the male-specific expression and meiosis-inhibiting action of Nanos2 in the germ cells of the fetal mouse testis [6], [30]. In contrast, we detected the opposite pattern of expression for *NANOS3*, which we find to be expressed at higher levels in fetal ovaries than in testes. *Nanos3* is expressed exclusively in pre-meiotic germ cells in the mouse and is downregulated shortly after the onset of meiosis [30], yet in the human fetal ovary we find the expression of *NANOS3* to increase significantly between 8–9 and 14–16 weeks gestation, concomitant with the onset of meiosis. This raises the intriguing possibility that NANOS3 may be involved in the regulation or progression of meiosis in human fetal ovarian germ cells; a finding that warrants further investigation. Our finding that *NANOS1* expression is maintained at a broadly constant level across the gestational range examined here is also unexpected, as NANOS1 protein is detectable only in the germ cells of the second trimester testis onwards [19]. The presence of *NANOS1* transcripts in the first trimester human fetal gonad suggests therefore that *NANOS1* mRNA may be subject to post-transcriptional regulation in human fetal germ cells.

We have demonstrated for the first time that RA can induce expression of *STRA8* in the human fetal testis. Utilizing a serum-free culture system used previously to investigate the effects of growth factor signalling in the human fetal ovary [31], we demonstrated increased *STRA8* expression in response to RA in cultures of human fetal testes at 14–15 weeks gestation – disaggregated to ensure germ cells were uncoupled from the local RA-metabolising action of neighbouring CYP26B1-expressing somatic cells. We failed to detect any changes in the levels of transcripts encoding the meiosis-specific proteins SYCP3 and DMC1 however, indicating that whilst RA is sufficient to induce *STRA8* expression, it cannot trigger widespread meiosis-associated gene expression in second trimester testicular germ cells. This may in part reflect our use of early second trimester tissues in this experiment, as expression of NANOS2, which acts to inhibit meiotic entry in testicular germ cells in a cell-intrinsic fashion [6], is at its peak at this time, or the relatively short (24 hours) period of culture used. Further experiments will need to be performed before conclusions as to whether RA can induce meiosis in the human fetal testis can be drawn.

In summary, we have characterized the expression of the essential components of RA signalling in the human fetal ovary and testis that may underpin the initiation of meiosis in the fetal ovary and its inhibition in the fetal testis. However, we identified key differences between humans and mice in the expression and distribution of components of the retinoic acid synthesis, signalling and effector machinery required for RA-regulation of sex-specific entry into meiosis. Whilst a key role for RA in the regulation of the initiation of meiosis in the human fetal ovary now appears indisputable [18], many aspects of this – particularly with respect to how this relates to the spatiotemporal organization of germ cell differentiation in the human fetal ovary and the apparent asynchronous entry of human fetal ovarian germ cells into meiosis – remain to be clarified. Detailed morphometric studies to establish the location of the first meiotic cells within the fetal ovary (aided by the development of antibodies to the human STRA8 and NANOS3 proteins) and the determination of the sites of retinoic acid synthesis and metabolism within the human fetal ovary and testis will be required to resolve these questions.

## Materials and Methods

### Ethics statement

Ethical approval for this study was obtained from Lothian Research Ethics Committee (study code LREC 08/S1101/1). All participants gave informed written consent in accordance with national guidelines [32].

### Collection of human fetal tissues

Human fetal testes and ovaries were obtained following elective termination of pregnancy during the first (50–65 days gestation) and second (13–19 weeks gestation) trimesters, as dated from last menstrual period. No terminations were for reasons of fetal abnormality and all fetuses appeared morphologically normal. Termination was induced with mifepristone (200 mg, orally) followed by misoprostol (Pharmacia, Surrey, UK) at 200 mg every 3 hours per vaginam. Gestational age was determined by ultrasound, and further confirmed by measurement of foot length for second trimester samples. The sex of first trimester fetal gonads was determined by PCR for the *SRY* gene as described previously [33]. Fetal gonads were carefully dissected in Dulbecco's Phosphate Balanced Salt Solution (DPBS; Invitrogen, Paisley, UK) and subsequently snap frozen and stored at −80°C for extraction of total RNA, fixed in Bouin's Solution and processed into wax by standard methods for immunohistochemistry, or prepared for culture as detailed below. Mesonephroi were carefully separated from first trimester gonads prior to RNA extraction.

### Culture of human fetal testes

Human fetal testes (14–15 weeks gestational age, n = 6) were mechanically and enzymatically disaggregated to yield a single cell suspension, as described previously for human fetal ovaries [31], [34]. Following washes with DPBS, the resulting cell suspension was divided in half, centrifuged at 1000 g for five minutes and pellets resuspended in serum free media (MEMα (Invitrogen), supplemented with 1× nonessential amino acids (Invitrogen), 2 mM L-Glutamine (Invitrogen), 2 mM sodium pyruvate (Sigma-Aldrich, Poole, UK), 3 mg/ml Bovine Serum Albumin (BSA; Sigma-Aldrich) and penicillin/streptomycin/amphotericin B (Cambrex Biosciences, MD, USA)) containing either vehicle (Dimethyl Sulfoxide (DMSO; Sigma-Aldrich); 0.1% final concentration), or 1 µM all-trans Retinoic Acid (Sigma; reconstituted in DMSO; 0.1% final concentration). Cells were cultured for 24 hours in a humidified incubator (37°C/5% CO_2_). After culture, adherent cells were lysed using buffer RTL (QIAGEN, Crawley, UK) and non-adherent cells collected by centrifugation and pellets re-suspended in buffer RTL. Lysates were pooled, and total RNA extracted as detailed below.

### RNA extraction and qRT-PCR

Total RNA was extracted from fetal gonads using the RNeasy Mini Kit (QIAGEN) with on-column DNaseI digestion as per the manufacturer's instructions. For the assessment of gene expression in non-cultured tissues, first strand cDNA was generated from total RNA using RT Kit (Applied Biosystems, Life Technologies, Carlsbad, CA) and real-time quantitative PCR (qRT-PCR) was performed using the Roche Universal Probe Library (Roche Applied Science, Burgess Hill, UK) on an ABI7900HT thermal cycler (Applied Biosystems) as described previously [35]. Primer sequences along with corresponding probe numbers are listed in Table S1. The expression level of each gene of interest was normalized to that of the 18S ribosomal RNA within the same sample. To determine the expression of STRA8, SYCP3 and DMC1 in cultured human fetal testis, first strand cDNA was prepared using the Superscript VILO mastermix kit (Invitrogen), and assessed by qRT-PCR using SYBR green technology on an ABI7500 Fast thermal cycler (Applied Biosystems) as described previously [33]. The expression level of each gene of interest was normalized to that of the housekeeping gene *RPL32* within the same sample. Primer sequences can be found in Table S2.

### Immunohistochemistry

Immunohistochemistry was performed on fixed sections of fetal ovary and testis tissue essentially as described previously [36]. Briefly, 5 µm thick sections of Bouin's-fixed, paraffin embedded tissues were mounted on electrostatically charged glass slides (BDH Chemicals, Poole, UK), dewaxed and rehydrated using xylene and graded alcohols and antigen retrieval performed by pressure cooking in 0.01 M sodium citrate buffer (pH 6) for five minutes. Endogenous peroxidase activity was blocked using 3% (w/v) hydrogen peroxide (H_2_O_2_) in methanol for 30 minutes, and slides were blocked using the avidin/biotin blocking kit (Vector Laboratories, Inc., Peterborough, UK) and incubation in Tris Buffered Saline (TBS), supplemented with 5% BSA and 20% normal serum (NS). Antibodies (listed in Table S3) were diluted in 5% BSA/TBS and applied to the sections at 4°C overnight. Antibodies were detected using the appropriate biotinylated secondary antibodies (30 minutes, 1∶500 dilution in BSA/TBS/NS) and incubation with avidin-biotin-HRP complex (Vector Laboratories). Bound antibodies were visualized using 3,3-diaminobenzidine tetrahydrochloride (DAB; DAKO, Cambridge, UK). Negative controls, incubated with blocking serum instead of primary antibody, were included in each experiment and displayed no staining (data not shown).

### Statistical analyses

Data presented represent mean ± standard error of the mean (SEM) of at least four independent biological replicates. Data were analysed using either ANOVA or Student's t test using Graphpad Prism Software. P values of less than 0.05 were considered statistically significant.

## Supporting Information

Table S1 **Oligonucleotide sequences and corresponding Roche Universal Probe Library numbers used in qRT-PCR assessment of gene expression in frozen human fetal tissues.**
(DOC)

Table S2 **Oligonucleotide primer sequences used in SYBR green qRT-PCR analysis of gene expression in cultured human fetal testes.**
(DOC)

Table S3 **Antibodies used for immunohistochemistry.**
(DOC)
