# Supplementary material for: Retinoic Acid Signalling and the Control of Meiotic Entry in the Human Fetal Gonad
Source: PLoS One. 2011 Jun 3;6(6):e20249. doi: 10.1371/journal.pone.0020249 (PMC3108594; doi:10.1371/journal.pone.0020249)
Supplement: Table S1 — Oligonucleotide sequences and corresponding Roche Universal Probe Library numbers used in qRT-PCR assessment of gene expression in frozen human fetal tissues. (DOC) [file pone.0020249.s001.doc]

| **Gene** | **Forward primer** | **Reverse primer** | **Probe** |
| --- | --- | --- | --- |
| *STRA8* | ggccttagctgtgcaaacac | ctcttcatcaacgggaaagg | 4 |
| *NANOS1* | tgaaagaaaaggtgcatttcaa | cctggctaagaaacattgtgc | 51 |
| *NANOS2* | gtcttcgcaggctcacct | ggcattgaaaggtgtcagc | 58 |
| *NANOS3* | gcagggttacttgtctctgtga | acaggtcaaaggtccccata | 44 |
| *RARα* | cagcaccagcttccagttagt | agaactgctgctctgggtct | 83 |
| *RARβ* | cacctgtcatcggaggactt | ggtgctctgtgtttcaattgtt | 16 |
| *RARγ* | cgctccatccaagagactg | gagggaactgggccgtag | 70 |
| *RXRα* | aagcggatcccacacttct | gaaggaggcgatgagcag | 18 |
| *RXRβ* | ggcggagaacaacaaacc | gtctgggcttcgggagtc | 36 |
| *RXRγ* | tgtcatgggcatgaagagg | cctcactctcagctct | 82 |
| *ALDH1A2* | ccacagtgttttccaacgtc | tcctgaacagggccaaag | 63 |
| *ALDH1A3* | ctggatgccctgagtcgt | ccctgtatccatcgtctcca | 22 |
| *CYP26A1* | gcagccacatctctgatcact | tgttgtcttgattgctcttgc | 45 |
| *CYP26B1* | acatccaccgcaacaagc | ggatcttgggcaggtaactct | 41 |
| *CYP26C1* | gccctcgacctaatcattca | gagctccacagccgactc | 17 |
