# Supplementary material for: Retinoic Acid Signalling and the Control of Meiotic Entry in the Human Fetal Gonad
Source: PLoS One. 2011 Jun 3;6(6):e20249. doi: 10.1371/journal.pone.0020249 (PMC3108594; doi:10.1371/journal.pone.0020249)
Supplement: Table S2 — Oligonucleotide primer sequences used in SYBR green qRT-PCR analysis of gene expression in cultured human fetal testes. (DOC) [file pone.0020249.s002.doc]

| **Gene** | **Forward primer** | **Reverse Primer** | **Ref** |
| --- | --- | --- | --- |
| *STRA8* | cctcaaagtggcaggttctgaa | tcctctaagctgcttgcatgc | [1] |
| *SYCP3* | agccgtctgtggaagatgag | caactccaactccttccagc | this paper |
| *DMC1* | agcagcaaagttccatgaag | tgagctctcctcttcccttt | [2] |
| *RPL32* | gcagggttacttgtctctgtga | acaggtcaaaggtccccata | [3] |

**Supplementary References**

1. Houmard B, Small C, Yang L, Naluai-Cecchini T, Cheng E, et al. (2009) Global gene expression in the human fetal testis and ovary. Biol Reprod 81: 438-443.

2. Kalejs M, Ivanov A, Plakhins G, Cragg MS, Emzinsh D, et al. (2006) Upregulation of meiosis-specific genes in lymphoma cell lines following genotoxic insult and induction of mitotic catastrophe. BMC Cancer 6: 6.

3. Coutts SM, Childs AJ, Fulton N, Collins C, Bayne RA, et al. (2008) Activin signals via SMAD2/3 between germ and somatic cells in the human fetal ovary and regulates kit ligand expression. Dev Biol 314: 189-199.
