# Supplementary material for: Retinoic Acid Signalling and the Control of Meiotic Entry in the Human Fetal Gonad
Source: PLoS One. 2011 Jun 3;6(6):e20249. doi: 10.1371/journal.pone.0020249 (PMC3108594; doi:10.1371/journal.pone.0020249)
Supplement: Table S3 — Antibodies used for immunohistochemistry. (DOC) [file pone.0020249.s003.doc]

| **Antigen** | **Species** | **Source** | **Antigen Retrieval** | **Dilution** |
| --- | --- | --- | --- | --- |
| RARα | Rabbit | Santa Cruz | No | 1.50 |
| RARβ | Rabbit | Abcam | Yes | 1.100 |
| RXRα | Rabbit | Santa Cruz | Yes | 1.100 |
